# A simple marking system for accurate intraoperative monitoring and adjustment of cyclotorsion strabismus surgery

**DOI:** 10.3389/fmed.2022.1059790

**Published:** 2023-01-06

**Authors:** Lung-Chi Lee, Hsu-Chieh Chang, Yi-Hao Chen, Ke-Hung Chien

**Affiliations:** ^1^Department of Ophthalmology, Tri-Service General Hospital, National Defense Medical Center, Taipei, Taiwan; ^2^Department of Nursing, Tri-Service General Hospital, National Defense Medical Center, Taipei, Taiwan; ^3^Graduate Institute of Nursing, College of Nursing, Taipei Medical University, Taipei, Taiwan

**Keywords:** cyclotorsion, disc-fovea angle, double Maddox rod, ocular torsion, torsional strabismus, surgical markings

## Abstract

Ocular cyclotorsion is treatable only with surgery. The surgical procedure must be tailored individually to the specific etiologies causing the horizontal and vertical strabismus and its torsional components. An adjustable surgical approach is often used for postoperative or intraoperative adjustments. However, the methods currently used have some limitations. In this study, we propose a simple intraoperative marking system for all cyclotorsion correction surgery. The proposed marking system used three sets of surface markers: external horizontal markings, ocular horizontal markings, and surgical torsion markings, drawn in sequence. We retrospectively analyzed the surgical results using this novel marking system in this single-center, single-surgeon study. Fifteen patients with cyclotorsion who underwent treatment using the proposed marking system as an intraoperative aid between August 2019 and August 2021 were included. The medical charts were thoroughly reviewed, and the pre-and postoperative subjective and objective cyclotorsion were analyzed. Among the study subjects (10 males, 5 females; age range: 6–89 years), 13 had excyclotorsion and 2 incyclotorsion. Preoperative mean net subjective cyclotorsion measured by the double Maddox rod (DMR) test was 6.0° (standard deviation: 10.8°) and mean net disc-to-fovea angle (DFA) was 20.23° (13.21°). The postoperative net DMR and DFA were 0.2° (2.1°) and 14.09° (5.97°), respectively. The mean absolute net DMR and DFA being treated were 9.8° (4.8°) and 9.76° (4.61°). Overall, the proposed intraoperative marking system is a simple and quantitative method to assess, monitor, and adjust the torsional aspect for all strabismus surgeries.

## 1. Introduction

Ocular torsion, or cyclodeviation, is the rotation of the eye along its anteroposterior axis, causing a torsional misalignment between the eyes in the primary position. Various disorders involving the cyclovertical extraocular muscles could result in cyclodeviation, including traumatic or ischemic superior oblique palsy (SOP), secondary strabismus caused by thyroid-associated orbitopathy, and skew deviation from brainstem lesions (1–3). Other possible causes are primary oblique muscle overaction with A- or V-pattern strabismus (4–6), inferior oblique paresis (7) or other vertical muscle palsies (8) from oculomotor nerve palsy, Brown syndrome (9), iatrogenic postoperative cyclotorsion from retrobulbar anesthesia (10, 11), and surgically induced cyclotorsion from macular translocation surgery (12), scleral buckling procedure (13), or consecutively from prior strabismus surgery (14–17). The resulting torsional misalignment may cause severe debilitating symptoms of cyclodiplopia to these patients in the acute state (18), which cannot be alleviated by any prism or orthoptic treatment, except for omitting vision entirely by occlusion or through sensory adaptation mechanisms (19) in chronic conditions. Surgical treatment is then the only possible means to restore correct vision.

The available surgical options vary based on the underlying specific disorder and anatomy. The surgical goals are achieved by targeting all six extraocular muscles and their pulleys to treat the causative vertical and/or horizontal deviations and the accompanied cyclotorsion, reducing all alterations (20). For excyclotorsion commonly caused by SOP, the oblique muscles are usually targeted through direct manipulation of the superior oblique (SO) muscles with strengthening procedures, such as SO tucking or the Harada-Ito technique (21), whereas the inferior oblique (IO) muscle can be weakened by myotomy or myectomy (22). In rarer cases of incyclotorsion, the SO can be treated with weakening procedures, such as tenectomy (23) or tendon spacer (24), or, more rarely, the IO strengthened with tucking or advancement techniques (25).

However, the resulting cyclo-alleviating effect of the same muscle procedure can vary greatly, and the outcome is often unpredictable with different etiologies and individual anatomical conformations (26, 27). Surgical techniques allowing postoperative (28–30) or intraoperative adjustments under local anesthesia (31) have been proposed. However, these modified techniques can be burdensome for young children and uncooperative adult patients, rendering them occasionally unsuitable. Herein, we propose a simple torsion marking system for intraoperative adjustment, providing an individualized, tailored, and targeted cyclotorsion surgery applicable to any procedure, which can be easily implemented under general anesthesia in all patients with torsional strabismus.

## 2. Materials and methods

### 2.1. Patients

This case series retrospectively analyzed patients with cyclotorsion who underwent strabismus surgery at the Ophthalmology Department of the Tri-Service General Hospital in Taipei, Taiwan between August 2019 and August 2021. The study protocol was approved by the Institutional Review Board (No: C202105113) of the Tri-Service General Hospital. The requirement for informed consent was waived by the review board according to the guidelines for a retrospective study. All patients were followed up for at least 6 months after the surgery.

The inclusion criteria were documented cyclotorsion and surgical treatment using the proposed technique. The data collected included demographic and clinical information, diagnosis, pre- and postoperative ophthalmic and strabismus examination results, the strabismus surgery performed, and pre- and postoperative fundus photographs. Patients with incomplete information were excluded from the study.

### 2.2. Examinations

**FIGURE 1:**
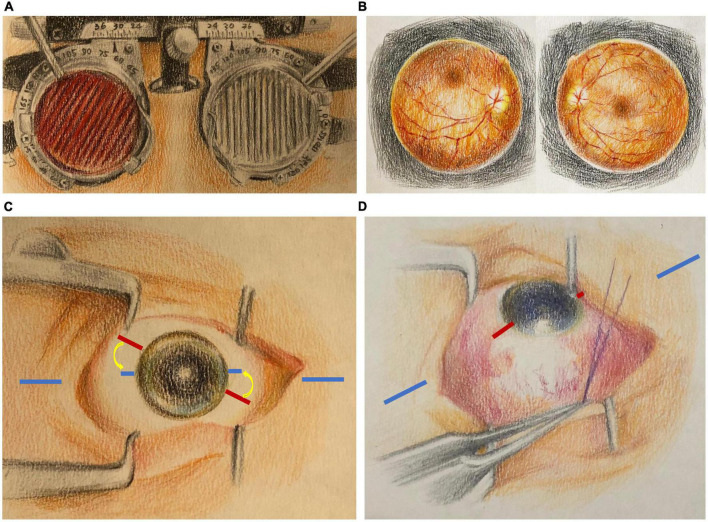
Pre- and intraoperative preparation for the simple torsion markings technique. In this instance, a significant incyclotorsion was measured subjectively with the double Maddox rod test **(A)** and confirmed objectively with the color fundus photography **(B)**. **(C)** The amount of cyclodeviation to be corrected was determined from the preoperative exams **(A,B)** and marked on the ocular surface before the surgery (red lines, indicating the surgical torsion markings) clockwise above the ocular and external horizontal markings (blue lines). For example, in a patient with a 15° incyclotorsion in the right eye, a surgical torsion marking should be marked 15°clockwise above the original ocular horizontal markings. In contrast, for a patient with excyclotorsion in the right eye, counter-clockwise surgical torsion markings should be placed below the original ocular horizontal markings, and vice versa with the left eye. **(D)** The surgical goal was to realign the surgical torsion markings (red lines) until parallel to the external horizontal markings (blue lines) using any adjustable surgical approach, which eventually achieved an excyclotorted correction.

The standard strabismus examinations included a prism alternate cover test to determine the angle of deviation in the primary and nine cardinal positions, a 3-step test, and a double Maddox rod test (DMR; Figure 1A), according to the protocol proposed by Liebermann et al. (32) to measure subjectively the cyclodeviation of the patient. Color fundus photographs (Figure 1B) were routinely acquired pre- and postoperatively to document the difference in the objective cyclodeviations, and the degree of the disc-fovea angle (DFA) was calculated using the online software Cyclocheck (33).

### 2.3. Surgical procedure

The procedure and surgical correction required were determined preoperatively by Dr. Chien, who performed all the strabismus surgeries under general anesthesia. All chosen surgical procedures were performed with an adjustable suture technique. The degree of cyclodeviation to correct was calculated based mainly on the subjective measurements from the DMR test, supported by the calculated objective DFA from color fundus photography (Figures 1A, B).

The proposed surgical technique for accurate intraoperative torsional adjustment is a simple marking system. First, the patient was asked to fixate directly ahead while staying in an upright head-straight position before inducing general anesthesia. With the guide of a simple toric reference marking device (Cionni Toric Reference Marker, Duckworth & Kent Ltd., Baldock, United Kingdom), the eye’s horizontal axis, parallel to the floor through the pupil, was marked using a marking pen able to withstand the disinfection procedures, preferably on the cornea, which is not affected during strabismus surgery. A horizontal line was also marked on the skin of the periocular region to add an external horizontal reference line for intraoperative adjustments. In this first step, the ocular horizontal markings and external horizontal markings should be aligned or parallel to each other. Second, the patient was placed in the supine position for anesthesia preparation and instructed to look straight ahead, alternatively with each eye if both eyes required surgery, to examine the ocular markings for significant cyclotorsion induced by postural change. If significant torsional change induced by the supine position was present, the ocular horizontal marking was updated to align or be parallel to the external horizontal markings. After the patient was anesthetized and properly draped, we ensured that the previous external and ocular horizontal markings were visible and adequate as a reference. Third, we marked the predetermined correcting degree for cyclodeviation on the ocular surface using a toric axis marking device (Cionni Toric Axis Marker, Duckworth & Kent Ltd.) with degree measurements (Figure 1C). Finally, the chosen strabismus surgery to correct any horizontal and/or vertical deviation and cyclodeviation was performed. Before the conclusion of the surgery, the surgical torsion markings were supposed to be aligned or parallel to the external horizontal markings. If the desired position was not achieved through the approach originally planned, adjustments were performed immediately to align the surgical torsion markings to the external horizontal markings (Figure 1D). Adjustments could be obtained with a modifiable suture technique or additional muscle surgery.

### 2.4. Main outcomes and statistical analysis

All postoperative data presented in Table 1 were obtained at 3 months postoperatively. Descriptive statistics were calculated using Microsoft Excel and expressed as mean (standard deviation, [SD]). The pre-and postoperative DMR and DFA were analyzed with their net value from the sum of the right and left eye.

**TABLE 1:** Summarized torsional characteristics.

No.	Age (years)/Sex	Diagnosis	Operation	Pre-op cover test	Post-op cover test	Pre-op DMR^†^ OD/OS (°)	Post-op DMR^†^ OD/OS (°)	Pre-op DFA^‡^ OD/OS (°)	Post-op DFA^‡^ OD/OS (°)	Overall corrected DMR (°)^§^	Overall corrected DFA (°)^¶^	Follow-up months
1*	49/F	Iatrogenic bilateral asymmetric CN3 palsy, consecutive torsional strabismus, OD	Multiple surgeries*	RET 14∧, RHoT 5∧*	RET 20∧, RHoT6∧*	−27/0	−4/0	−20.54/5.58	0.40/5.18	23	20.54	23.4
2	88/M	SOP, ischemic, OD	Recession of IO to the IR insertion with anterior transposition, OD	RHT 4∧	Ortho	5/2	−1/2	4.55/5.95	−0.15/6.58	−6	−4.07	9.3
3	6/F	Alternating esotropia with primary IOOA, OS > OD status post prior strabismus surgery with residual IOOA, OS > OD	Recession of IO (4 mm), OD and posterior displacement of IO (4 mm posterior to IR), OS	IOOA (OS 2+ > OD 1+); right head tilt	No more head tilt and IOOA	−3/13	−1/0	0.19/22.47	1.23/9.21	−11	−12.22	6.5
4	89/M	SOP, ischemic, OS	Recession of SR (2 mm) + disinsertion of IO, OS	LHT 16∧; 3-step: LRL	Ortho	0/12	−1/−2	8.30/15.66	5.96/2.62	−15	−15.38	6.2
5	51/M	SOP, ischemic, OD	Recession of IO inferiorly (4 mm) and medially (2 mm), OD	RHT 2∧; 3-step: RLR	Ortho	5/0	−1/0	6.43/11.22	5.56/6.42	−6	−5.64	9.4
6	57/M	Consecutive hypertropia, OS	Recession of IO (4 mm), OS	LHT 2∧; 3-step: negative	LHoT 1∧	1/6	1/0	11.37/15.40	10.23/9.67	−6	−6.87	9.7
7	6/M	Congenital SOP with secondary IOOA, OS status post prior strabismus surgery with residual left hypertropia with IOOA and right head tilt	IO myotomy + recession SR (2 mm), OS	LHT 6∧; IOOA 3 + (OS); right head tilt	Ortho but still mild right head tilt; IOOA 2+ (OD, reversal)	1/4	0/−1	12.26/10.41	8.91/7.31	−6	−6.45	10.2
8	63/F	SOP, ischemic, OS	Recession of SR (8 mm), OS	RHoT 25∧; 3-step: LRL	Ortho	0/12	0/0	7.99/20.45	7.64/8.34	−12	−12.46	6.2
9	66/M	Idiopathic orbital inflammation with secondary hypertropia, OD	Recession of SR (7 mm) + muscle biopsy, OD	RHT 30∧	Ortho at primary; ET 8∧ with LHoT 6∧ at down gaze	−7/−1	−1/0	−2.86/2.26	1.45/4.61	7	6.66	12.1
10	49/F	Alternating exotropia with left hypotropia and strong right eye preference	Recession of IR (5 mm), OS	LXT 6∧, LHoT 15∧	Ortho	4/8	2/0	14.84/17.28	12.35/10.24	−10	−9.53	6.2
11	58/F	TAO, OU with secondary hypertropia and esotropia, OD	Recession of IR (10 mm) and MR (6 mm), OD	RHoT 30∧, RET 40∧; mild limitation of upper gaze (OD)	Ortho	8/0	1/0	16.54/7.82	9.13/7.14	−7	−8.09	8.3
12	19/M	Traumatic SOP with secondary IOOA, OD	Resection of IO (8 mm), OD	RHT 8∧; IOOA 1.5+ (OD); 3-step: RLR	Ortho	12/0	2/0	7.08/12.53	−2.9/13.34	−10	−9.17	6.1
13	27/M	Congenital SOP with IOOA, OS and right head tilt	Recession of IO + recession of SR (1.5 mm), OS	LHT 4∧; IOOA 3 + (OD); right head tilt,	Ortho	13/5	4/0	22.47/13.71	12.38/8.30	−14	−15.5	9.6
14	73/M	SOP, ischemic, OS with VR surgery for RD 3 months prior	Recession of IO (10 mm) + recession SR (3 mm), OS	LHT 4-6∧	Ortho	0/12	0/3	8.19/22.54	8.48/13.52	−9	−8.73	15.4
15	7/M	Congenital SOP with secondary IOOA, OS	Recession of IO (10 mm), OS	LHT 4∧; IOOA2 + (OS)	Ortho	0/5	0/0	9.32/14.07	9.09/9.15	−5	−5.15	6.7

CN, cranial nerve; DFA, disc-center-fovea angle; DMR, double Maddox rod; ET, esotropia; HoT, hypotropia; HT, hypertropia; IO, inferior oblique; IOOA, inferior oblique overaction; IR, inferior rectus; MR, medial rectus; OD, right eye; OS, left eye; OU, both eyes; RD, retinal detachment; TAO, thyroid-associated orbitopathy; SOP, superior oblique palsy; SR, superior rectus; VR, vitreoretinal; XT, exotropia.

^†^Pre-and postoperative cyclotorsions were measured with the subjective double Maddox rod test and were the main factor determining the surgical markings. Positive numbers indicate excyclotorsion and negative numbers incyclotorsion.

^‡^The pre-and postoperative DFA was calculated using the online software Cyclocheck and used to confirm the subjective torsion measured.

^§^The difference between the pre-and postoperative DMR sum of the right and left eye was calculated to represent overall correction.

^¶^The difference between the pre-and postoperative DFA sum of the right and left eye was calculated to represent overall correction.

*Patient 1 had multiple surgeries and was presented in detail in the text.

## 3. Results

### 3.1. Main outcomes

Fifteen patients (10 male and 5 female) met the inclusion criteria and underwent surgery with the proposed marking system for torsion correction between August 2019 and August 2021. The mean (SD) age was 47.29 (28.15) years, and three patients were under 18 years (Patients 3, 7, and 15), whereas the oldest was 89 years old. Etiologies contributing to the torsional aspect of the strabismus included ischemic (*n* = 5) or traumatic (*n* = 1) trochlear nerve palsy, congenital SOP with inferior oblique overaction (IOOA) (*n* = 2), primary IOOA (*n* = 2), thyroid-associated orbitopathy (*n* = 1), and other types of strabismus (*n* = 4). The surgical approach for each case differed according to the underlying etiologies; the clinical presentations are listed in Table 1.

There were 13 cases of net exyclotorsion and 2 of net incyclotorison preoperatively. The preoperative DMR ranged from 36° of incyclotorsion to 13° of excyclotorsion. The overall mean (SD) of the preoperative net DMR was 6.0° (10.8°), and the mean (SD) DMR was 0.8° (9.3°) for the right eye and 5.2° (5.1°) for the left eye, respectively. The preoperative overall mean (SD) net DFA was 20.23° (13.21°), and the mean (SD) DFA was 7.08° (9.84°) for the right eye and 13.16° (6.13°) for the left eye. The overall mean (SD) of the postoperative net DMR was 0.2° (2.1°), and the mean (SD) DMR was 0.1° (1.8°) for the right eye and 0.1° (1.1°) for the left eye. The postoperative mean (SD) net DFA was 14.09° (5.97°), and the mean (SD) DFA was 5.98° (4.84°) and 8.11° (2.91°) for the right and left eyes, respectively. The mean absolute net DMR and DFA being treated were 9.8° (4.8°) and 9.76° (4.61°), respectively. Patients 1, 9, and 15 are discussed here to illustrate the simplicity and the advantages of this marking system for intraoperative adjustment to correct the torsion degree simultaneously with other surgical treatments, such as for vertical strabismus or IOOA.

### 3.2. Case presentation

**FIGURE 2:**
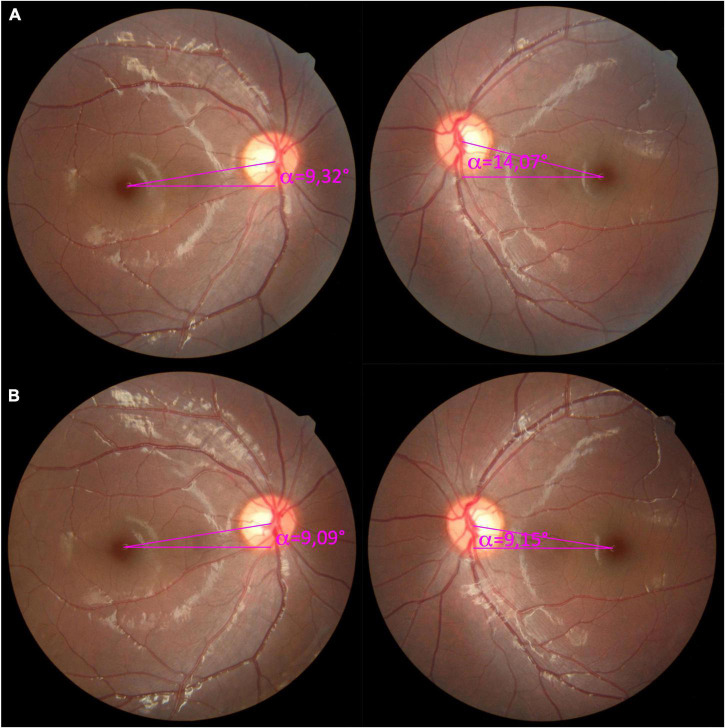
Pre- and postoperative color fundus photographs for Patient 15. **(A)** The preoperative color fundus revealed a disc-fovea angle (DFA) of 9.32° in the right eye and 14.07° in the left eye, with 5° of excyclotorsion in the left eye measured with the double Maddox rod (DMR) test. **(B)** At the postoperative 3-month follow-up, the DFA was 9.09° in the right eye and 9.15° in the left eye with no subjective torsion at the DMR test.

Patient 15 was a 7-year-old boy with a significant right head tilt. Left hypertropia with 4 prism diopters and grade-2 IOOA were observed, and the 3-step test was positive for left SOP. The DMR test revealed subjective excyclotorsion of 5° in the left eye only. The objective calculated DFA from color fundus photography using Cyclocheck was 9.32° in the right eye and 14.07° in the left eye, for an overall 4.75° excyclotorsion in the left eye, consistent with the result of the subjective DMR test. Hence, a surgical 5° incyclotorsional effect was predetermined for surgical torsion marking. A 10-mm IO recession was performed to correct the primary vertical deviation, IOOA, and excyclotorsion, using the surgical markings described in the Section “Materials and methods.” Intraoperatively, the IO muscle was disinserted and repositioned on the sclera at the intended 10-mm recession position, then temporarily tied only with a slip knot for possible adjustments. The surgical effect was assessed to determine whether the recession was sufficient to align the surgical torsion marking with the external horizontal markings. The markings were aligned, and the suture was then tied securely to complete the surgery. The 3-month postoperative strabismus examination revealed orthotropia with grade-1 IOOA and no subjective torsion on DMR testing which remained stable throughout follow-up. The DFA calculated with Cyclocheck in the left color fundus photograph revealed a reduction in cyclotorsion to 4.57°compared to the preoperative DFA (Figures 2A, B).

**FIGURE 3:**
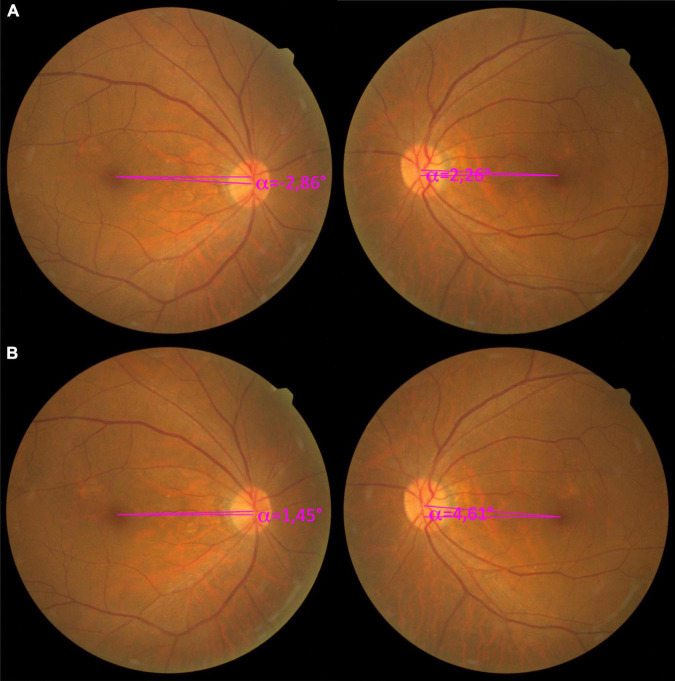
Pre- and postoperative color fundus photography of Patient 9, with a right superior rectus muscle hypertrophy due to idiopathic orbital inflammation. **(A)** The preoperative color fundus photograph showed a relatively small disc-fovea angle (DFA) in both eyes with an easily missed incyclotorsion of the right eye, with a −2.86° DFA, and a 2.26° DFA in the left eye. However, the subjective net incyclotorsion was 8° measured by the double Maddox rod (DMR) test. **(B)** The 3-month follow-up fundus photograph showed a net reduction of 4.31° excyclotorsional effect from the superior rectus recession, with the right DFA being 1.45° and the left DFA 4.61°.

Patient 9 was a 66-year-old male with acute vertical, and subsequent torsional, diplopia. Initial strabismus examination revealed 8 prism diopters of right hypertropia and 6 prism diopters esotropia were noted. The 3-step test was inconclusive. A diagnosis of idiopathic orbital inflammation with right superior rectus muscle hypertrophy, causing secondary strabismus was made. Medical treatment with systemic steroids was prescribed. During the treatment and observation period of 6 months, the disease was relatively quiescent, and the strabismus stabilized on a right hypertropia of 20–25 prism diopters at the simultaneous prism cover test with fusion, and 30 prism diopters on the DMR test. However, a net right incyclotorsion of 8° was also revealed by the DMR test, with symptoms under a corrected prism. The preoperative DFA was −2.86° in the right eye and 2.26° in the left eye (Figure 3A). A strabismus surgical correction was then planned with superior rectus muscle recession, using the hang-back technique, and muscle biopsy for tissue proof. The proposed torsion marking system was applied intraoperatively to monitor the torsional effect of the muscle biopsy combined with the corrective muscle-weakening procedure. Eventually, the surgical torsion marking was set approximately 8°clockwise to the external horizontal markings to correct 8° of incyclotorsion. At the 3-month follow-up, the DFA was 1.45° in the right eye and 4.61° in the left eye (Figure 3B), and the patient was orthotropic in the primary position with 1° subjective incyclotorsion in the right eye, with an incomitant esotropia and left hypotropia on downward gaze.

**FIGURE 4:**
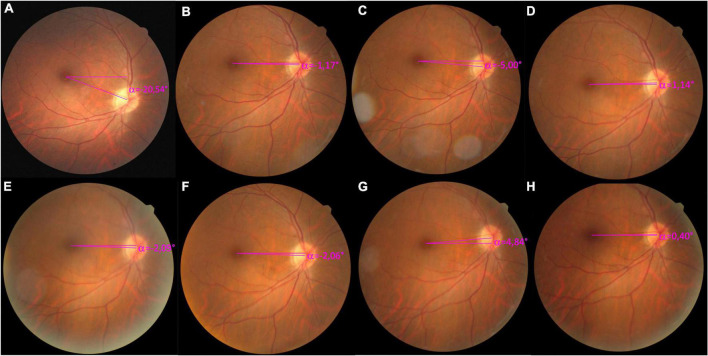
Serial objective cyclotorsion changes of the right eye for Patient 1 depicted by color fundus photography. **(A)** Photograph acquired before the first attempted surgery with the proposed marking system, with 27° of incyclotorsion measured with the double Maddox rod (DMR) test and a −20.54° of disc-fovea angle (DFA). **(B)** The 1-month postoperative results of the first surgery showed a marked improvement with the DMR test showing 3° of incyclotorsion and a markedly reduced DFA (−1.17°). **(C)** Five months after the first reoperation, a residual subjective incyclotorsion of 10° with a −5° DFA was noted. A second surgical procedure was then performed. **(D)** One month after the second surgery. **(E)** Two months after the second surgery. **(F)** Three months after the second surgery, a residual of 10° incyclotorsion measured with the DMR test was noted. A third procedure of scar revision and transposition of scar and residual muscle was performed to correct the torsion. **(G)** One month after the fifth reoperation. **(H)** Five months after the fifth reoperation. The DMR test revealed a 4° of incyclotorsion and the DFA was 0.40°.

Patient 1 was a 49-year-old woman who suffered from intraoperative bleeding during endoscopic transnasal transphenoid removal of an invasive pituitary macroadenoma with clival involvement 5 years earlier. Postoperatively, the patient experienced a bilateral asymmetric oculomotor nerve palsy with secondary strabismus. Multiple strabismus surgeries were attempted to treat the different aspects of the ever-evolving secondary strabismus, from a marked exotropia to a consecutive esotropia, with evident right hypotropia, and a significant 40° incyclotorsion due to secondary SO overaction. However, previous attempts failed to correct the debilitating symptomatic cyclodiplopia. The patient was then referred to Dr. Chien with a constant right esotropia, hypotropia, and significant incyclotorsion measuring 27° on DMR testing and −20.54° DFA on the color fundus photographs (Figure 4A). Owing to the severe conjunctival scarring, the surgical plan was devised after meticulous dissection of the conjunctival scar tissue. Scarred muscle and scar tissue complexes were identified on the eyeball at several attachment sites and detached, then secured with 6-0 Vicryl (Ethicon, Raritan, NJ, USA) for adjustment. The surgical torsion markings were targeted for an excyclotorsion effect of 30°, marked clockwise above the horizontal reference line. Eventually, a transposition of the medial rectus muscle to the lateral side of the original inferior rectus muscle insertion was performed, with transposition of the inferior rectus muscle to the inferior side of the lateral rectus muscle. The 1-month postoperative result was temporarily satisfactory with 3° of incyclotorsion on DMR testing and −1.17° of DFA (Figure 4B). Unfortunately, after 5 months, the incyclotorsion increased to 10° on DMR testing with −5.00° DFA (Figure 4C). A second operation with transposition of the superior and lateral rectus muscle insertion was performed to correct the residual torsion. The intraoperative torsion correction was targeted to 10° excyclotorsion effect using the proposed surgical torsion marking system; however, due to the poor muscle tone and severe scarring noticed intraoperatively, the outcome was unsatisfactory, with a residual 5-10° incyclotorsion on DMR testing and −1.04° to −2.09° DFA (Figures 4D–F). After 3 months we attempted another reoperation. The residual muscles and scar tissues were temporarily fixated and adjusted solely based on the effect that could align the surgical torsion markings to the external horizontal markings. The primary surgical aim to correct the symptomatic cyclodiplopia and treat the residual horizontal and vertical strabismus using a prism was achieved. The patient had no residual cyclodiplopia one month postoperatively (Figure 4G). At the 5-month follow-up, the patient had a 4° incyclotorsion on DMR testing and 0.40° DFA, with constant right esotropia and hypotropia corrected by prism (Figure 4H). The patient was symptom-free with fusion and satisfied with the surgical outcome.

## 4. Discussion

We presented a simple marking system to monitor and adjust the torsional effect with ease while treating cyclodeviation during strabismus surgery. The proposed marking system does not require specific additional tools in the operating room, only a marking pen and toric marking devices for routine astigmatic correction. The markings are drawn in three parts: first, a cutaneous horizontal marking for external reference; then, an ocular horizontal marking to indicate the supine torsional effect and guide to mark for the predetermined surgical correction; and finally, a surgical torsion marking showing the required torsional correction. The proposed marking system follows a sequence of simple steps to ensure accuracy and consistency and can be applied to all types of cyclotropia and implemented in any procedure to correct, assess, or monitor the surgical torsional effect. Using this marking system during the correction of cyclotorsion strabismus, we demonstrated an overall mean absolute net 9.8° (4.8°) subjective cyclotorsion correction, achieving satisfactory results of an average of net DMR 0.2° (2.1°) postoperatively.

The success of a cyclotorsion correction is determined by the preoperative assessment and the surgery itself. Hence, an adequate and accurate cyclotorsion quantification is paramount. Cyclotorsion measurement includes objective and subjective methods. Objective methods measure the anatomical torsion, defined as the position of the fovea relative to the optic disc, classically quantified using the nominal Guyton’s grading system with an indirect ophthalmoscope or fundus photography (34). Other techniques involve using the retinal vascular arcades (35), retinal temporal raphe (36), iris recognition (37, 38), and scleral blood vessels (39). A more accurate numerical measurement most accepted by clinicians, the DFA, is defined as the angle between a horizontal line and a line drawn through the fovea and the center of the optic disc. The DFA can be measured using fundus photography (40, 41), perimetry (42), scanning laser ophthalmoscopy (43), or optical coherence tomography (44, 45). Anatomic variations of the DFA in normal subjects can range from −0.4° to 12.76°, with a mean of 6.39° (2.72°), and the angle is typically slightly larger in the left than in the right eye (46). Furthermore, because of sensory adaptation, ocular dominance, anomalous retinal response, and other mechanisms, the objective measurement may differ from the subjective torsional perception (19, 47, 48).

Subjective methods to measure cyclotorsion include the Bagolini striated lens test (49), single (50) and double Maddox rod (DMR) test (32), synoptophore test (51), and Lancaster red-green test (52). However, these methods have different procedural protocols and may yield different results (53, 54). Furthermore, while subjective cyclotorsion in healthy subjects is relatively low, around 1° of excyclotorsion, the cyclofusional amplitude can range from 7° of incyclotorsion to 9° of excyclotorsion (55). Hence, the motor-defective cyclovergence accounting for the symptomatic cyclodisparity should be the main treatment target when determining the degree of surgical correction required to alleviate the cyclodiplopia since the intended corrective influence of a stress-lessened cyclofusion and other sensory neuroadaptation mechanisms are usually still active. Accordingly, the subjective DMR test was chosen in our study as the main tool for treatment assessment for its reliability and repeatability, low cost, wide availability, and dissociative nature (32).

Cyclotorsion correctional surgery is not a standardized procedure and is usually planned based on the individual’s cyclovertical manifestation and the surgeon’s preference. Surgery usually warrants some modifications or adjustments to the individual anatomy or pathology to achieve a satisfactory result. The most common approach is to adopt an adjustable suture technique and adjust the correction postoperatively within a day based on a subjective measure, such as a DMR test (28, 29). Intraoperative one-stage adjustment under topical anesthesia had been proposed by Xie et al. achieved positive results (31); however, this approach is challenging in children or uncooperative adult patients. Furthermore, the patient is usually required to be in an upright position for the subjective measurement either under local anesthesia or general anesthesia with an intraoperative awake phase (56), which poses additional challenges.

Hence, as strabismus surgery is usually performed under general anesthesia, intraoperative adjustments must be monitored using objective methods, namely, observation of the fundus torsion using an indirect ophthalmoscope (24, 27). However, this presents some challenges. First, intraoperative fundus rotation evaluation is a subjective skill requiring considerable surgical experience, with differences among surgeons. Second, a relative head tilt of the patient or surgeon while performing the indirect ophthalmoscopy would complicate the assessment (57). Moreover, the supine position in general anesthesia induces 5° or more postural cyclotorsion in either direction in over 40% of these patients, confounding the results (58, 59). Furthermore, the objective anatomical torsion often differs from the subjective target cyclotropia correction; and lastly, the method used to measure the cyclotorsion should be the same in the assessment process before, during, and after surgery, for consistent results.

Therefore, an intraoperative torsion monitoring method that can be performed under general anesthesia without the deficiencies of the objective indirect ophthalmoscopy, such as the proposed marking system, has great potential. Previous studies proposed using limbal markings at the 12 and 6 o’clock positions in complicated multiple-muscle surgery to monitor the torsional effect, with great success (60, 61). Our proposed method has three sets of markings: an ocular marking placed horizontally with the patient in upright position, which can be adjusted in supine position while alternating fixation in case of posture-induced ocular torsion; an added external horizontal marking on the skin for standard reference, together with the ocular marking serve as the corrective guide for exotropia eye position often seen in deep general anesthesia; and surgical torsion markings, showing the subjective correction required directly on the ocular surface rather than on the fundus. Hence, the surgeon can further quantitatively monitor, assess, and adjust the torsional effect during any procedure with ease and accuracy.

Our study has some limitations. First, the markings may be altered or deleted by scrub solution, intraoperative heme, or other factors. Hence, other techniques using VERION Image Guided System (Alcon Laboratories, Ft. Worth, TX, USA) or other iris recognition systems can be used to adapt this technique to microscope oculars for augmented reality viewing during strabismus surgery (62). Second, in patients with combined large-angle exodeviations or vertical deviations, the two markings on the ocular surface may be difficult to assess under general anesthesia, which resumes both eyes into their resting position farther apart. Hence, the second ocular horizontal reference marking should be drawn carefully, and the patient should be instructed to fixate alternately with each eye looking straight forward while sitting upright and in the supine position before anesthesia. This ensures the accuracy of the ocular horizontal reference as the base for the surgical torsion markings in these significantly deviated eyes. Then, using a new method proposed by Fu et al., the immediate target endpoint at the conclusion of the surgery can be assessed (63). Third, the sample size was relatively small; however, our results demonstrate the utility of the proposed marking system in several clinical scenarios. Further research should involve larger and more diverse groups.

In conclusion, the proposed marking system can be used in all types of strabismus surgery at any age and for any presentation, under local or general anesthesia, allowing the correction of subjective cyclotorsion and posture-induced ocular torsion. This simple system applies basic techniques and modalities to provide quantitative intraoperative assessment and guide adjustments during cyclotorsion correction and can be utilized by all strabismus surgeons at any level of experience.

## Data availability statement

The original contributions presented in this study are included in the article/Supplementary material, further inquiries can be directed to the corresponding author.

## Ethics statement

The studies involving human participants were reviewed and approved by Institutional Review Board (No: C202105113) of the Tri-Service General Hospital. Written informed consent from the participants’ legal guardian/next of kin was not required to participate in this study in accordance with the national legislation and the institutional requirements.

## Author contributions

K-HC: conceptualization, methodology, writing—review and editing, and funding acquisition. L-CL, H-CC, and K-HC: formal analysis and investigation. L-CL: writing—original draft preparation. Y-HC and K-HC: resources and supervision. All authors read and agreed to the published version of the manuscript.
