# Supplementary figures and images for: A simple marking system for accurate intraoperative monitoring and adjustment of cyclotorsion strabismus surgery
Source: Front Med (Lausanne). 2023 Jan 6;9:1059790. doi: 10.3389/fmed.2022.1059790 (PMC9853205; doi:10.3389/fmed.2022.1059790)

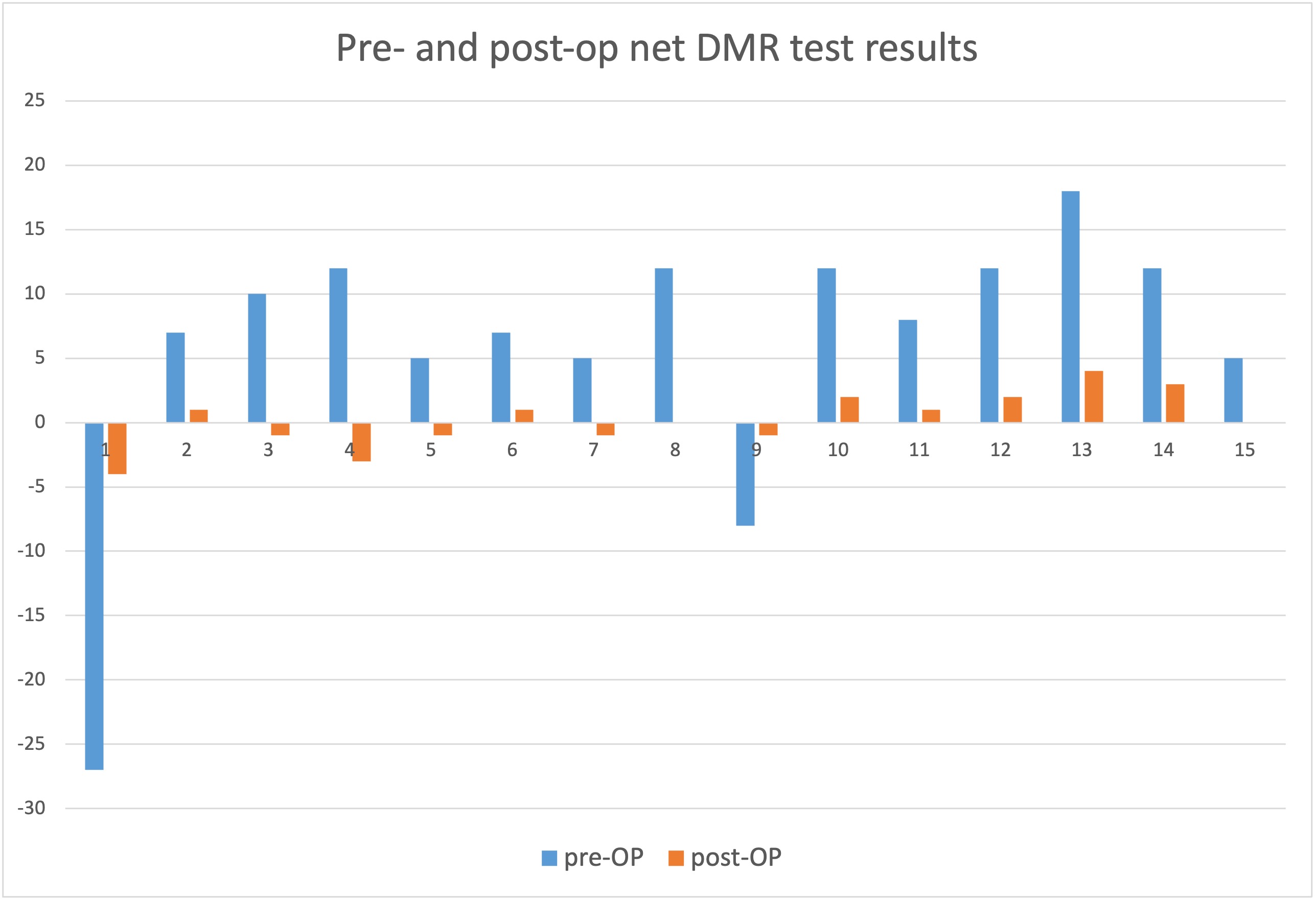

Supplement: Supplementary file 1 [file Image_1.JPEG]

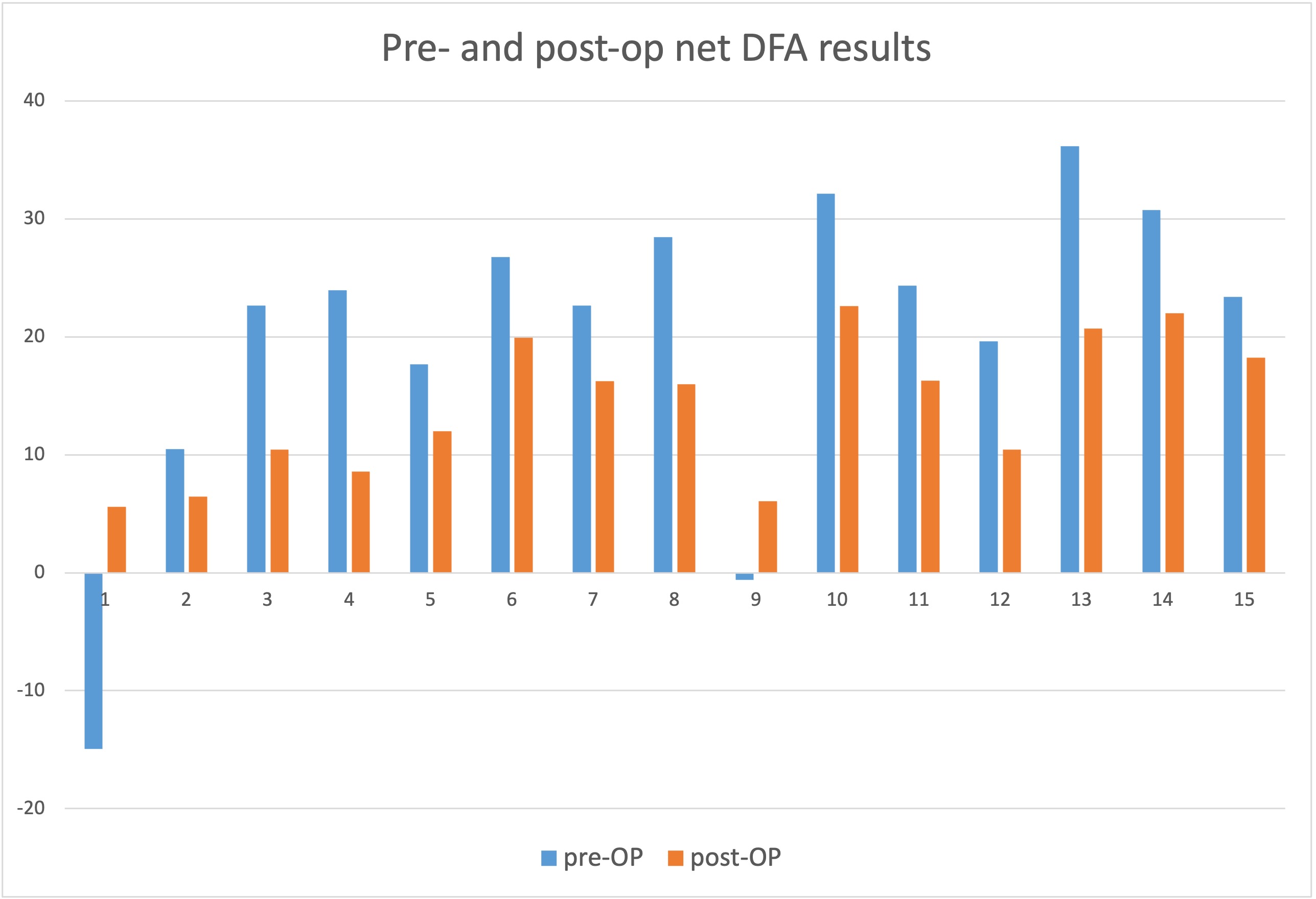

Supplement: Supplementary file 2 [file Image_2.JPEG]
